# Barriers in implementing the dying patient law: the Israeli experience - a qualitative study

**DOI:** 10.1186/s12910-020-00564-5

**Published:** 2020-12-11

**Authors:** Avi Zigdon, Rachel Nissanholtz-Gannot

**Affiliations:** 1 Department of Health Systems Management, School of Health Sciences, Ariel University, Science Park, P.O.B. 3, 4070000 Ariel, Israel; 2 Smokler Center of Health Policy Research, Myers-JDC-Brookdale Institute, P.O.B. 3886, 91037 Jerusalem, Israel

**Keywords:** End-of-life, Palliative care, Physician, Law

## Abstract

**Background:**

Coping with end-of-life issues is a major challenge for governments and health systems. Despite progress in legislation, many barriers exist to its full implementation. This study is aimed at identifying these end-of-life barriers in relation to Israel.

**Methods:**

Qualitative in-depth interviews using professionals and decision makers in the health-care and related systems (*n* = 37) were carried out, along with two focus groups based on brainstorming techniques consisting of nurses (*n* = 10) and social workers (*n* = 10). Data was managed and analyzed using Naralyzer software.

**Results:**

Qualitative analysis showed identification of six primary barriers: 1) law, procedures, and forms; 2) clinical aspects; 3) human aspects; 4) knowledge and skills of medical teams; 5) communication; and 6) resource allocation. These were further divided into 44 sub area barriers.

**Conclusions:**

This study highlights the role of the family doctor in end-of-life by training physicians in decision-making workshops and increasing their knowledge in the field of palliative medicine. Effectively channeling resources, knowledge, and support for medical teams, by accounting for the structure and response of the units for home treatment will improve patient’s access to information on and support for end-of-life laws, as well as reduce legislative barriers in other countries that face the same issues.

## Background

Coping with patients at the end of their lives is a major issue for governments and health systems in the developed world. The approach whereby every person has the right to die with dignity, without forfeiting his autonomy and the right to make decisions about his life and death, is now accepted in Western countries [1, 2]. Nonetheless, many patients struggle with the fear of slow death and a situation where they will not be able to express their opinion on the question of which medical treatments they wish to receive, or not receive, at the end of their lives [3, 4]. Different countries reach a different balance between patient autonomy and other values [5]. Even at the individual level, differences of opinion may arise among family members and between family members and medical staff, and frequently these involve no information about the patient’s wishes regarding end-of-life treatment [6]. Conversations with patients for end-of-life planning are associated with fewer aggressive interventions and with improved quality of life at the end of life [7]. In addition, it is linked to lower levels of depression, tension, and anxiety as well as higher levels of satisfaction in the patient’s family after death [7, 8]. In fact, the presence of written directives is correlated with less tension among family members after the patient dies [9]. On the other hand, there is also evidence that writing advance healthcare directives is not sufficient to ensure end-of-life treatment that complies with the patient’s wishes [10]. In recent years, many countries have passed laws that stipulate both rules for end-of-life treatment as well as the status of the patient’s pre-stipulated wishes in a situation of incompetence at the end of life [11, 12].

However, many doctors prefer that the patient will raise the issue [13, 14], partly because of lack of time or training. The difficulties of communication among family members, and not only with the medical establishment [15], do not always allow for decision-making that is based on the patient’s personal free will [16, 17]. Hence, there is a need to teach and educate medical teams to talk with their patients about end of life issues and help them fulfill instructions regarding their wishes [18]. Models have even been developed for integrating social workers into the patient’s end-of-life decision-making process [19], and advanced reflective simulation exercises assist in improving medical staff proficiency in handling End-of-Life situations [20].

In 2005, The Dying Patient Law was enacted in Israel, which is an example of how the State of Israel deals with these issues. However, despite its enactment 15 years ago, its implementation has proven faulty. The law is based on the values of the State of Israel as a democratic state and takes into account a variety of religious and moral concepts (section 1 (b) of the law) [21]. In formulating this legislation, various ethical values were taken into account, including: the principle of the sanctity of life, the quality of life principle, the principle of prevention of pain and suffering, and the principle of autonomy.

Prior to the enactment of the dying patient law, there were only a few court cases dealing with dying patients [22–24]. The relevant law was the Patients Rights’ Law – 1996, which states that every treatment must be accompanied by informed consent, on the one hand, and if a person is in danger of death he must be treated even without consent. There were diverse interpretations in a situation when someone was unable to give a consent at the end of his life and it wasn’t clear what his wish was or even if we knew he did not want to be treated, it was not clear whether he should be treated [25]. The Dying Patient Law includes the right to refrain from receiving medical treatment. The uniqueness of the law is that it allows a person to avoid, at will, life-prolonging medical treatment and to give these instructions in advance, in case he is unable to express his desire not to receive life-prolonging treatment. The law also states that every effort should be made to alleviate the pain and suffering of a dying patient [21].

Along with the desire to allow the patient to make end-of-life decisions, there are quite a few barriers to the implementation of laws dealing with the end of life. These include lack of time to discuss the subject, a difficult and cumbersome medical form containing advance healthcare directives, and the basic responsibility of doctors to act to save and prolong life [26]. From the patient’s perspective, it was found that educated, less religious elderly people are more interested than others in being involved in decision-making at the end of their lives [27]. Open communication and reflective listening are essential in examining the barriers in the various issues.

## Methods

The aims of the study are to identify and map the barriers to the implementation of legislation relating to the dying patient in Israel from the viewpoint of healthcare teams and leaders in the Israeli health system in order to propose solutions for reducing legislative barriers as well as share the Israeli experience with other countries who face these issues. Despite progress in dealing with the issue and the very existence of legislation in Israel and in other Western countries, many barriers exist to implementing such legislation in the Western world, and not only in Israel.

### Study design and data collection

The study is comprised of two major sections. Part 1 includes 37 in-depth interviews with professionals and decision makers on the Dying Patient Law in the health system, including 14 Health Maintenance Organization (HMO) representatives (3 family physicians, 2 home care physicians, 2 home care nurses, 6 administrative workers, and a hospice physician), 11 general hospital representatives (an ICU chief physician, a geriatric care division director, a geriatric department chief physician, a radiation department chief physician, 2 social workers, 2 nurses, an ethical advisor and a general physician), 6 assisted living and retirement home representatives, 2 ministry of health representatives, 2 hospices representatives, and 2 End-of-Life association representatives. Part 2 includes two focus-groups comprised of nurses and social workers. Each section is based on a different research tool. In both parts, the interviewees were codified using letter “N” (Nurse), “M” (Manager), “FGSW” (Focus Group Social Workers), “FGN” (Focus Group Nurse), “P” (Physician), “SW” (Social-Worker), “H” (Hospice) and “PA” (Patient Advocacy) followed by an ordinary number.

### Part 1 - in-depth interviews with professionals and decision makers

We conducted in-depth interviews with 37 professionals and decision makers who are engaged with the issue of the dying patient in the health care system. (Table 1 presents the Characteristics of Sample In-depth interviews). The interviews were conducted face to face and lasted between 45 and 75 min.

**Table 1:** Characteristics of Sample - In-depth interviews

	Male	Female	Total
**HMO representatives**			14
Family doctors	3		
Doctors in homecare units	2		
Nurses in homecare units	1	1	
Doctor in home hospice unit		1	
Staff workers	2	4	
**Representatives of general hospitals**			11
Doctors at administrative level	4	1	
Social workers		3	
Nurses		2	
Ethics advisors (ethics expert)		1	
**Representatives of old age homes and assisted living**			6
Administrative positions	3	1	
Nurses		1	
Social worker		1	
**Health Ministry representatives**			2
Staff workers involved in design, implementation, and supervision of the law	2		
**Hospice representatives**			2
Owner of company supplying home hospice services (physician)	1		
Director of institutional hospice (physician)		1	
**NGOs and private organizations**			2
Jurist and ethics expert		1	
Social worker	1		
**TOTAL**	19	18	37

The interviewees were selected using the “snowball” method, in order to reach a diverse sample of interviewees from various levels (field personnel, managers of treatment units, and policy makers in staff positions), professions (doctors, nurses, social workers, and experts in law and ethics), and institutions (health-funds, hospitals, hospices and nursing homes, representatives of related associations, and Ministry of Health representatives). At the beginning of each interview, we asked the interviewees’ consent to record the interview. The taped interviews were transcribed, with two exceptions where the interviewees refused to be recorded. The interviews were conducted by means of an interview manual, which uses guiding questions, but enables a conversation that can develop in different directions. This method allows the researcher to remain attentive to the research subject and the unique issues raised, on the one hand, while not forgetting to ask about substantive issues related to the research topic [28]. The interview manual places a different emphasis on each interview, depending on the issues relevant to the interviewee’s position and the institution to which the interviewee belongs.

All the interviewees were asked about issues relating to the Dying Patient Law: How the law affected their work; actions taken at the institution where they work in order to implement the law; degree of awareness of the law and the subject of end-of-life treatment in general; objections they encounter regarding the law and dealing with end-of-life treatment in general; their perception of the extent to which the law is applied; possible reasons for non-implementation or incomplete implementation of the law; their perception of the importance and necessity of the law; changes that have taken place in end-of-life treatment in recent years (not necessarily as a result of the law); and ways to improve the response to patients at the end of their lives, in terms of the quality of the treatment and exercising their right to decide on how they will be treated.

### Part two – focus groups

We held two focus groups, one of nurses and the other of social-workers. The representatives were sent by the authorities in the health funds or in the nursing homes. Each of the focus groups had 10 participants. The group of nurses included 10 female nurses and the social workers group included 8 women and 2 men, from the health funds and from institutions for seniors. (Table 2 presents the Characteristics of Sample: Focus Groups).

**Table 2:** Characteristics of Sample - Focus Groups

	Focus group Nurses	Focus group Social workers	Total
**Clalit Health Maintenance Organizations**	3	1	4
Nurses	3		
Social workers		1	
**Maccabi Health Maintenance Organizations**	2	3	5
Nurses	2		
Social workers		3	
**Meuchedet Health Maintenance Organizations**	2	3	5
Nurses	2		
Social workers		3	
**Leumit Health Maintenance Organizations**	1	1	2
Nurses	1		
Social workers		1	
**Facilities for senior citizens**	2	2	4
Nurses	2		
Social workers		2	
**TOTAL**	10	10	20

The focus groups were conducted using brainstorming techniques [29]. The questions were open-ended, and the researchers made sure that they received feedback from each of the participants. The discussions in the focus groups were recorded and summarized by one of the researchers. Each focus group was presented with six main questions during the instruction phase: 1) How do you define successful treatment of a patient at the end of life; 2) What difficulties do you experience during a patient’s end-of-life treatment; 3) What is your organization’s attitude to the subject of holding conversations with the patient to clarify preferences at the end of life; 4) How would you like to see the treatment of patients at the end of their lives conducted by your organization; 5) What would help you or the system achieve the situation you described; and 6) Have you witnessed any change over the past few years in end-of-life treatment?

### Qualitative data analysis

In order to identify all the barriers to implementing the law, the study findings were analyzed using Naralyzer, the qualitative analysis software, which enables content analysis by building category trees that bring together all the contents relating to a particular category but distinguishes between the different groups of interviewees. The qualitative analysis procedure in this study is based on the grounded theory. This theory makes it possible to conceptualize qualitative data from texts to understand the subject under study. In the first phase of data analysis, the text was disassembled from the interviews and focus groups into “meaning units”. Each meaning unit constituted a passage from the text, stood on its own and served as a cornerstone of the analysis. In the second stage, the meaning units were labeled with specific codes that represent ideas/concepts that have a similar meaning and are included in the same conceptual framework. The first and second stages formed the basis for the categorical division of the codes obtained. In the third stage, the categories that make up the theoretical structure of the analysis were constructed. Each category was constructed based on common general ideas of codes tagged in the second stage with possible connections. Several codes with a common conceptual framework were combined to create a category.

This method enabled us to identify the barriers to end-of-life legislation, those that emerged from the in-depth interviews, and those that emerged from the focus groups.

## Results

**Fig. 1:**
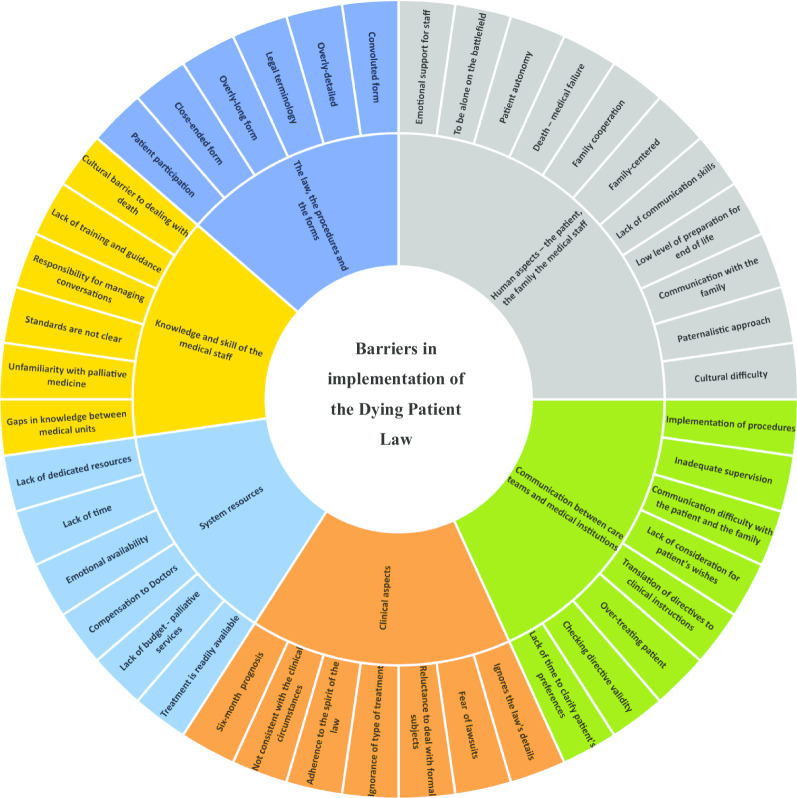
Barriers in the implementation of the Dying Patient Law

This study found that there are still many barriers to the implementation of the law, which are expressed in six main themes: The law, procedures, and forms; Human aspects of the patient, the family, and the medical staff; Knowledge and skills of the medical teams; System resources; Clinical aspects; and Communication between clinicians and medical organizations. In these six themes, we identified 44 areas that generate these barriers as reflected in the Results section. (See Fig. 1).

### Barriers stemming from the law, procedures, and forms

The form which the patient is asked to sign constitutes one of the main obstacles to the implementation of the Dying Patient Law in the State of Israel. The form is written in legal language and is not comprehensible to the average person; it is too long and full of details, which promotes mistakes in filling it out, making it hard for the patient to participate in the signing process. A homecare nurse expressed frustration with the forms thus: *“The ones who wrote the form are lawyers, where covering for themselves was the most important part for them, not the people [...] who fill it out with the patients.”* [FGN4].

Specifically, the excessive details regarding the case and the definition of suffering is seen by many of the participants as unnecessary and burdening. Thus, care-takers think that the legal emphasis and the lack of ability to express the patient and his family’s opinions, these forms miss their target, even when intended to fill an important role in their interpretation in the moment of truth. *“It misses the target […] a structured form has advantages, but the biggest disadvantage is […] that it doesn’t allow for personal expression […] they need to do something that gives place to the patient and his family, and that way we can make decisions that fit better with their point of view”* [N1].

Even after receiving complete and detailed medical explanations, the patient does not have the capability to understand the complete meaning of the medical procedures described in the form. *“The form contains a lot of difficult and burdening questions, and even if the patient receives the best possible explanation for the medical terms from the best doctor, he will still have a hard time answering the questions”* [M7].

For clinicians, therefore, legal emphasis and failure to allow for expression of patient opinion mean that the forms fall short of achieving their goal. Even after receiving comprehensive medical explanations, the individual rarely has the necessary ability to understand the full significance of the medical treatments listed in the form. As one manager noted: *“The form has a lot of demanding and difficult questions, and even if the patient receives the best explanation from the best doctor who will explain the medical terms to him, he will have trouble answering the questions.”* [M7] On the one hand, according to the social workers, the fact that clarifying the patient’s preferences and following the directives are not a regular part of the protocol of a home visit impedes the implementation of the law: ***“****We have to make a very clear protocol routine: A, B, C, D [...], and you have to mark the X in the right place.”* [FGSW7] On the other hand, there was opposition to this assertion in the nurses’ focus group, with their opinion being that clinicians should be left with the flexibility to clarify these issues in a manner that is timely and appropriate for each one of them: *“The most important thing is to open up a space, to let you talk about what you would like and what’s important to you and what your preferences are.”* [FGN4].

### Barriers arising from clinical aspects

Most interviewees opposed the use of a six-month life expectancy definition because this measure could not be used for every type of disease. While in certain types of cancer, there are metrics by which the patient’s life expectancy can be estimated, in other diseases there is difficulty in predicting life expectancy and it cannot be defined as 6 months, a fact that prevents the patient from falling under the scope of the law.

For patients who are not defined as “dying patients” and therefore are not covered by the law, no preliminary-medical-guidelines can be implemented. In cases where their wishes are unknown and they are unable to express them, the assumption is that they would like to live and should be given treatment. This presumption causes difficulty and great resentment among many care teams. In their opinion, the law clashes with accepted professional views. In the words of one manager: ***“****They put the staff in an impossible situation [...] Help the patient and you’re a criminal, or else force upon the patient treatment that an hour ago, a day ago, a week ago, he told you he did not want.”* [M3] It should be noted that according to Israeli law, the medical staff can fulfil and respect previous wishes of the patient, including a wish to avoid medical treatment. Although there was great support for the spirit of the law and the principles it charts, there was still criticism and frustration that the details of the law do not match the clinical reality.

*“Jurists can say ‘that is right, that is wrong” by moral or legislative categories, but in a large portions the law is in disagreement with reality […] so either there is no legal answer, or there is an answer and it does not fit with our professional views“* [P9]. In many cases, the end-of-life patient has many diseases, and it is not clear which of these diseases will result in the patient’s death. Thus, physicians avoid setting and documenting the patient’s life expectancy because of the uncertainty involved, in addition to the fear of being sued using the information given in these forms. *“A problem I encountered is many physicians are not willing to sign the instructions: I will not sign that the patient understood all that I’ve explained, and that is why the instruction is understanding-based”* [M5].

### Barriers arising from human aspects - patient, family and medical staff

Implementing the Dying Patient Law creates personal and cultural barriers for the staff, the patient, and the family, as well as a cultural difficulty in dealing with death. Some interviewees noted that many physicians still take a paternalistic approach, and their perception of whether to refrain from treatment due to futility or to extend life is a professional decision made by the physician alone. However, it was noted that there has been progress in doctors’ perceptions, and in recent years awareness has been increasing, especially among younger doctors. The older the patients and the poorer their cognitive state, the less awareness they have of their rights, with the medical staff less disposed to speak to them directly. Most of the communication in these situations is focused on the family, even if the patient is competent. As one social worker put it: *“When I come in at the stage of home care, I feel a sense of having missed out because I did not know the patient before [...] I know that the hospital will determine that he is not competent.”* [SW3] It also emerges that the percentage of citizens prepared for the end of life is very low, and if the patient did not do early preparatory work while he was healthy or in the non-hazardous stages of his illness, it will be much harder to start thinking about end of life in the stages when death is approaching. As one nurse noted: *“I really see that patients are dodging the possibility that ‘maybe I will not survive’ [...] It is very threatening.”* [N1] In practice, people from different cultures will not necessarily agree on whether it is right to tell the patient everything about their condition, and, in cases of disagreement, the clinicians do not know how to act. The patient’s family plays a very significant part in the treatment process at the end of life. Hence, when the patient is in the early stages of the disease or is still healthy, it is important to promote communication within the family on the issue. However, it is acknowledged that encouraging communication is not a feasible solution for every family, and, in any event, a way must be found to deal with cases where there is a conflict between family and patient wishes.

### Barriers in the knowledge and skills of the medical teams

Some of the most significant barriers to the implementation of the law raised in the study relate to the lack of knowledge and skills among the clinical staff. Almost all of the interviewers noted that most of the staff lacked the complex proficiency needed for holding painful conversations with end-of-life patients. In addition, a cultural barrier, especially among doctors, makes it difficult to cope with the subject of death. Many noted that there is no awareness of the obligation imposed by law on the medical staff in all matters relating to informing the patient of his condition and exploring his preferences.

In fact, there is no clear statement about who should be responsible for initiating conversations with patients towards the end-of-life and raise this issue. The unclear issue of responsibility creates ambiguity, which touches not only upon the family and hospital doctors, but also the experts in the community, home care units, and nursing homes. A physician said: *“The loop between the care givers is not closed, and everyone says, ‘Why should I do it? Let him/her do it!’ But what is right?”* [P1] The difficulty of establishing rules on the subject came up in the interviews, since the right person to conduct the conversation depends very much on the specific situation, and especially on the type of illness. As one social worker noted: *“The question of who will manage this issue-the community or the hospital-depends on the illness”* [SW4]. Moreover, a number of palliative experts noted that there is a difficulty since the Ministry of Health has not established clear standards for palliative care, and this undermines the quality of treatment and the equality of access. Some of the participants in the study think that nursing homes and assisted living facilities are actually an ideal place for palliative care outside the hospital.

### Barriers in communication between care teams and medical institutions

When a patient from the community is admitted to the hospital, the medical staff is required to clarify his preferences and check if his advanced directives are valid and relevant. Unfortunately, the staff is not always available to do it. There is adherence to this process in relevant situations, such as person who suffers from widespread malignancy and there is danger to his life or an oncologist patient who is beyond treatment. This situation has been in this research as a barrier to fulfilling the patient’s will. Some of the interviewees noted the importance of translating the directives into specific clinical instructions for dying patients, such as the Physician Orders for Life-Sustaining Treatment (POLST) form used in the US. Others noted that the solution should be to improve end-of-life services in the community so that patients who are receiving only palliative treatment will not even be taken to a hospital in an emergency.

In nursing homes, it also emerged that an unequivocal demand made of the Health Ministry is essential in order to have the matter of the advance directives implemented. As one manager said: *“In institutions we operate according to the procedures of the Ministry of Health. That is what the Ministry of Health stipulates! If they don’t ask, we don’t act.”* [M1] The interviews reveal that involving the management, especially hospital managers and department managers, is critical to the successful implementation of the law and palliative care: *“From inside the hospital management, it is possible to bring about change. It is our responsibility [...] There are priorities, and this was not one of them. The director of the hospital, and not the nursing director, should have led this.”*[P8].

The focus groups also clearly demonstrated the need to implement the issue from the top-down –the health ministry directorate and the health funds, through the directors of the wards to the staff members themselves - in order to move beyond local initiatives. Nevertheless, the management may prove to be a barrier: *“The social workers and the nurses in the field were happy to go in this direction, but even here the administrative factors are a barrier. They are very afraid that things will not be done exactly according to the forms and the law, and then they will not be covered.”* [P9] When staff members talk about end-of-life options, they sometimes encounter resistance because they fail to make it clear that acknowledging the end-of-life and the cessation of aggressive therapies does not mean not abandoning the patients.

### Barriers in resource allocation

The enactment of the law was not accompanied by the addition of dedicated resources to the organizations which are responsible for its implementation. Conversations to clarify preferences and instructions take a long time, especially for a patient with complex care needs. The problem is not only allocating the time, but also the emotional availability such conversations require. Physicians that we interviewed stated that it was impossible to hold such a conversation during the frenzied pace of a normal working day, when patients were waiting outside: *“How can I even talk to them about ‘what would you like?’ Do I have to go to their home? Not to do it in the clinic?”* [P12] The issue of compensation appears to be secondary compared to the issue of time, and if the doctors had time for it and were convinced that this was correct medicine, there would be no need for specific compensation, just as there is none for other types of treatment. In the words of one nurse: *“Once a chronic disease is detected [...] it takes time, skill, a lot of listening. Unfortunately, this does not happen. It happens more with patients with severe complex care needs.”* [N6] Another aspect of lack of resources is lack of additional budgets earmarked for the expansion of palliative services, with an emphasis on low of standards for experts and those responsible for implementation. Moreover, insufficient funds can detrimentally affect training and mentoring of teams: *“Everything that the Ministry of Health decides and is not earmarked specifically in the budget, such as the guidelines regarding palliative medicine [...] is always problematic. And then everyone improvises [...] just in order to discharge their minimal obligation.”* [P10] In the health funds, there is a growing awareness for palliative care at home. All health funds have established Home-Hospice-Units that specialize in palliative care and available 24/7 for planned and urgent home visits. The teams of these units include a professional palliative multi-disciplinary team. The service is provided by HMO teams or by teams of private companies from which the HMOs purchase the service, and the costs are higher. The number of people who can get the service is limited.

## Discussion

The issue of dying patients is often difficult and painful, provoking ethical, moral, ideological, and religious disputes. This study sheds light on the difficulties and many obstacles to the implementation of decisions regarding the end of life. Such laws cause the same difficulties to arise, whether or not there is a cultural understanding of their necessity- respecting the wishes of an individual at the end of life. Implementing and applying the legal instructions create many challenges stemming from impediments in the broader setting where the actual implementation of the law takes place. This study shows that there has been significant progress in terms of the perception of the “dying patient,” of the individual expressing his will and having it honored. The health system teams are more aware of the patient’s wishes and understand the importance of upholding them. The change that has occurred in this area may be the result of, among other things, a change in attitude in the medical world, from a paternalistic to an autonomic approach, in which the doctor knows better what is right for the patient and makes the decision for him, to an autonomous approach emphasizing individual freedom and recognizing the patient’s right to decide what treatment to get (if any) based on non-medical considerations. Even if the study participants had criticism of the details of the law, the general attitude was that the law has done important work in raising awareness of the issue and in urging medical bodies to take action. In addition, it legitimized practices that preceded the legislation and clarified legal and ethical issues. At the same time, the law made it easier for physicians to document medical guidelines for withholding treatment, which they feared to do before the law was passed.

Other research data indicated barriers that were reflected in the difficulty in discussing the subject with patients and families [26]. This study shows that the law can address this barrier, opening up the conversation possibilities on sensitive end-of-life matters. The literature further shows that physicians do not agree with legislation that permits physician-assisted suicide [30], which is forbidden in Israel according to the law and apprehensions were voiced about the doctors’ function in disconnecting patients from life-support devices [13]. Indeed, the issue of the central role of the physician was also raised in this study and in many countries around the world. However, until otherwise established, the one who determines the handling ​​of the dying patient, as reflected in the legislation, is still the doctor. In England [31], the physician has the authority to refuse a patient’s request that treatment be continued if he believes that the treatment is not helping the patient. This is in contrast to Israel [21], where the physician is required to continue to treat the patient who wishes it, even if there is no medical justification for doing so.

One of the obstacles mentioned is unawareness of the details of the law, which creates a gap between the rights granted by law and the implementation of these rights. This gap requires proactive measures to increase awareness of the subject of end-of-life planning. One of the ideas that was raised in the study is that directing consciousness-raising action or thinking can be achieved by educating the public, in general, and talks with family members of patients. This does not mean forcing the use of these rights, but becoming familiar with the legislation and creating an awareness among the public, emphasizing that recognizing the basic right of a person to plan the end of his life is unavoidable. The role of the medical system should be to provide all relevant medical information and inform patients that the option of the advanced directives exists. But the patient’s coping with emotional consequences and uncertainties as well as the continuation of the discussion with the family will be carried out with other factors, more oriented to social and spiritual dimensions. Training physicians, and in particular recruiting senior physicians to implement the subject through workshops on decision-making, communication, and end-of-life treatment, may contribute to increasing awareness and promoting the engagement of the entire medical staff. As part of the solutions proposed by the interviewees in this study, it was found that it is important for physicians in the units caring for dying patients to become more knowledgeable about the field of palliative medicine, so that they can provide basic palliative care without having to refer to specialists in the field.

The issue of the “products of the law” arose in most of the interviews. According to the interviewees, the forms should be simplified and made more user-friendly. As one specialist in patient advocacy noted, one constructive step would be *“to simplify the form and turn it from the nine-page form it is today to a maximum of two to two-and-a-half pages.”* [PA1].

One of the main problems is that the form is a part of the law. This means that to change the form and make it more user friendly, requires a new law and not just MOH directives.

In the last few years, many participants have detected more sensitivity to the existence of patient rights and more awareness that it is not right to give treatment at all costs. However, the study participants also noted that the barriers raised are not necessarily related to the law, but rather to parallel and separate progress in the clinical approach to end-of-life quality treatment.

This issue, of course, is not restricted to Israel. Most western countries are concerned with the extent to which speeding up end of life should be permitted in societies that, in general, do not support suicide. However, in most countries, including Israel, attempted suicide is no longer considered a criminal offense. One of the reasons for the difficulty in solving the end-of-life issue is the question of whether an individual has the right to end his life. A discourse of rights implies the counterbalancing notion of obligations. Thus, recognition of a person’s right to terminate his life as he wishes raises the issue of the obligation of society to assist him in doing so. Such a societal obligation runs contrary to the received views of care teams, and physicians in particular, as well as the perception that the right to life is one that cannot be relinquished. That is, a person given life cannot decide to “waive” it. The prevailing view is that the right to life is very basic to, and in the fabric of, individual rights. As such, it is important not only to the individual, but also to society.

## Limitations

This study, as all others, has limitations that stem from the methods used. Qualitative in-depth interviews with interviewees that play a central role in an organization represent the organization’s opinion and not that of the public. In this research patients were not sampled or interviewed, and the focus group of nurses and social workers represent only the opinion of medical staffs.

## Conclusions

In Israel and the western world, the relationship between clinician-care teams and patients is strong. Medical teams in the community are in daily contact with elderly and sick populations. However, the proportionate increase in the elderly population of developed economies such as Israel in relation to the number of medical teams will, in the not-too distant future, strain care team access to this demographic. This will thus affect implementation of laws dealing with end-of-life. Therefore, it is recommended that the role of the family doctor in end-of-life treatment should be strengthened. The structure and response of the units for home treatment should be taken into account, to enable greater accessibility to homes for the aged, medical assistance housing, and hospice homes that can provide appropriate response to end-of-life patient needs. At the same time, awareness must be raised among the general population, medical staff as well as other therapists in the health system. This is needed to channel the resources, knowledge, support, and tools to these medical teams for improving treatment and responding to patients who need information and support for mastering end-of-life laws, with the aim of promoting the legitimate rights of all citizens who are at the end of their lives.

## Data Availability

Data sharing is not applicable to this article as no datasets were generated or analyzed during the current study.

## Abbreviations

HMO: Health Maintenance Organization
NGOs: Non-Governmental Organizations
MOH: Ministry of Health
N: Nurse
M: Manager
FGSW: Focus Group Social Workers
FGN: Focus Group Nurse
P: Physician
SW: Social-Worker
H: Hospice
PA: Patient Advocacy
